# Role of the IL-23/IL-17 Pathway in Rheumatic Diseases: An Overview

**DOI:** 10.3389/fimmu.2021.637829

**Published:** 2021-02-22

**Authors:** Claudia Schinocca, Chiara Rizzo, Serena Fasano, Giulia Grasso, Lidia La Barbera, Francesco Ciccia, Giuliana Guggino

**Affiliations:** ^1^Rheumatology Section, Department of Health Promotion, Mother and Child Care, Internal Medicine and Medical Specialties, University Hospital “P. Giaccone”, Palermo, Italy; ^2^Department of Precision Medicine, University of Campania “Luigi Vanvitelli”, Naples, Italy

**Keywords:** IL-23, IL-17, IL-23/IL-17 axis, inflammatory diseases, autoimmune diseases

## Abstract

Interleukin-23 (IL-23) is a pro-inflammatory cytokine composed of two subunits, IL-23A (p19) and IL-12/23B (p40), the latter shared with Interleukin-12 (IL-12). IL-23 is mainly produced by macrophages and dendritic cells, in response to exogenous or endogenous signals, and drives the differentiation and activation of T helper 17 (Th17) cells with subsequent production of IL-17A, IL-17F, IL-6, IL-22, and tumor necrosis factor α (TNF-α). Although IL-23 plays a pivotal role in the protective immune response to bacterial and fungal infections, its dysregulation has been shown to exacerbate chronic immune-mediated inflammation. Well-established experimental data support the concept that IL-23/IL-17 axis activation contributes to the development of several inflammatory diseases, such as PsA, Psoriasis, Psoriatic Arthritis; AS, Ankylosing Spondylitis; IBD, Inflammatory Bowel Disease; RA, Rheumatoid Arthritis; SS, Sjogren Syndrome; MS, Multiple Sclerosis. As a result, emerging clinical studies have focused on the blockade of this pathogenic axis as a promising therapeutic target in several autoimmune disorders; nevertheless, a greater understanding of its contribution still requires further investigation. This review aims to elucidate the most recent studies and literature data on the pathogenetic role of IL-23 and Th17 cells in inflammatory rheumatic diseases.

## Review

### Interleukin-23

Interleukin-23 (IL-23) is a member of the IL-12 cytokine family composed of the IL-23p19 subunit and the IL-12/23p40 subunit, the latter shared with IL-12, encoded by genes located on chromosomes 12q13.2 and 11q1.3, respectively (1–3).

IL- 23 is mainly secreted by activated macrophages and dendritic cells (DCs) located in peripheral tissues, such as skin, intestinal mucosa, joints and lungs (4–6).

Despite the protective role played by the IL-23/IL-17 axis against bacterial and fungal infections, extensive knowledge supports the contribution of its dysregulation in triggering chronic inflammation and autoimmunity, providing a solid substrate for the development of several autoimmune diseases like PsA, Psoriasis, Psoriatic Arthritis; AS, Ankylosing Spondylitis; IBD, Inflammatory Bowel Disease; RA, Rheumatoid Arthritis; SS, Sjogren Syndrome; MS, Multiple Sclerosis (3, 7–10) (Table 1).

**Table 1:** Therapeutic agents in rheumatic diseases.

	**SpA**	**PsA**	**SS**	**SLE**	**RA**	**Target**
Secukinumab	x	x	Ongoing trials	Ongoing trials	Ongoing trials	IL-17a
Ixekizumab	x	x			Ongoing trials	IL-17a
Brodalumab	Not approved				Not approved	IL-17R
Bimekizumab	Ongoing trials				Ongoing trials	IL-17a, IL-17f
Netakimab	Ongoing trials					IL-17a
Ustekinumab	Not approved	x		Ongoing trials		IL-12, IL-23
Guselkumab	Not approved	x			Not approved	IL-23
Apremilast	Not approved	x				PDE4
Tofacitinib	Ongoing trials	x			x	JAK1-JAK3 (JAK2)
Baricitinib					x	JAK1-JAK2
Upatacitinib	Ongoing trials				x	JAK1
Filgotinib	Ongoing trials					JAK1
Rituximab			Off label	Off label	x	CD-20
Tocilizumab			Not approved		x	IL-6R

*SpA, Spondyloarthritis; PsA, Psoriatic Arthritis; SS, Sjogren Syndrome, SLE, Systemic Lupus Erhytematosus; RA, Rheumatoid Arthritis*.

The main role of IL-23 is to induce the differentiation of αβ T CD4^+^ naïve cells (Th0 cells) in T helper type 17 (Th17 cells) (11, 12), which are considered pivotal players in autoimmunity (1, 9).

**Figure 1:**
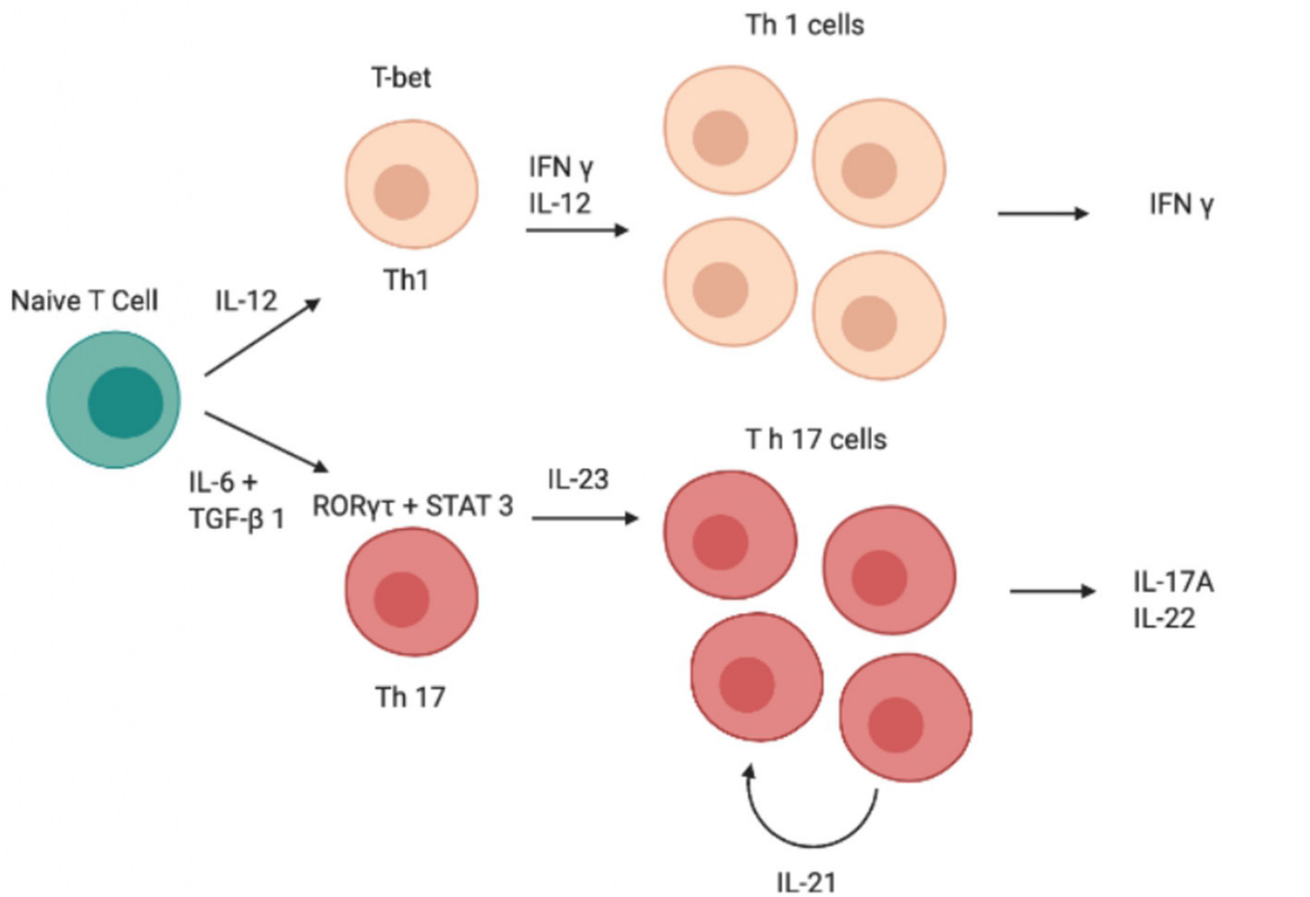
Schematic image of the cascade of cytokines and transcription factors involved in the differentiation of Th1 and Th17 cells. IFN-γ, interferon-γ; IL, interleukin; RORγt, retinoid-related orphan receptor γt, STAT, signal transducer and activator of transcription; GATA, GATA transcription factor; TGF-β, transforming growth factor β, Th, T helper.

Although IL-12 and IL-23 are both members of the IL-12 family and have a similar structure, the role of these two cytokines in Th0 differentiation is totally different (13, 14); indeed, unlike IL-23, IL-12 induces differentiation of Th0 cells into T helper type 1 (Th1 cells) rather than into Th17 (Figure 1) (15–19). Both cytokines are produced by DCs and the balance of IL-12 and IL-23 production is controlled by prostaglandin E2 (PGE2), which promotes inflammatory responses (20, 21).

IL-12 and IL-23 act as a bridge between the innate and adaptive arms of the immune response (22). IL-12, produced by antigen presenting cells (APCs), is essential for the optimal proliferation and production of cytokines by Th1 cells in response to antigens. Overall, IL-12-induced IFN-γ is an effective activator of the antimicrobial functions of phagocytes and plays a critical role in resistance to many pathogenic bacteria, fungi and intracellular parasites (23).

Regarding the IL-23/IL-17 axis, γδ T cells and innate lymphoid cells (ILCs) constitutively express the IL-23 receptor (IL-23R), suggesting their prompt first-line response to IL-23, followed by cytokine secretion and subsequent activation of the adaptive immune response. Moreover, since Th0 cells do not express IL-23R, they require prior stimulation with transforming growth factor β (TGF-β), IL-6 and IL-21 to become responsive to IL-23 (24–29).

In response to IL-23-mediated activation, αβ T cells, γδ T cells and ILCs produce IL-17, IL-22, TNF-α, and interferon-gamma (IFN-γ); in addition, IL-23-activated γδ T cells make αβ T cells refractory to the suppressive activity of regulatory T cells (Treg) and they also prevent the conversion of conventional T cells into FOXP3+ Treg cells *in vivo* (30).

### IL-23 Receptor

The IL-23 receptor (IL-23R) is a heterodimeric receptor composed of 2 subunits: IL-12Rβ1, in common with the IL-12 receptor (IL-12R) and IL-23Rα, specific to IL-23 signaling (31).

Therefore, T cells lacking IL-12Rβ1 cannot respond to IL-12 nor IL-23. Conversely, IL-23Rα-deficient T cells cannot respond to IL-23, while maintaining IL-12 signaling capability (32). IL-23Rα and IL-12Rβ1 chains are expressed on T cells, natural killer (NK) T cells, monocytes/macrophage and DCs (33).

The intracellular pathways require different signaling proteins: JAK2, Janus kinase 2; TYK2, tyrosine kinase 2; STAT3, STAT4, signal transducer and activator of transcription 3 and 4 (34, 35).

Specifically, IL-12Rβ1 binds to TYK2 inducing STAT4 phosphorylation which is essential for increasing IFN-γ production and subsequent Th1 cells differentiation. Instead, IL-23Rα interacts with JAK2, inducing STAT3 phosphorylation and leading to the upregulation of retinoid-related orphan receptor gamma tau (RORγt), crucial for the development of Th17 cells (19, 31, 36, 37).

Once activated, STATs homodimers translocate into the nucleus, where they bind to DNA in the promoter region of target genes, acting as downstream effectors in the IL-23/IL-12 signaling pathway (38, 39).

Finally, the evaluation of IL-23 functions *in vivo* in different mouse models supports the hypothesis that IL-23 may act in both lymph nodes and peripheral tissues to drive terminal differentiation of effector Th17 cells *in vivo*, promoting Th-17-mediated inflammation. Confirming these observations, in the absence of Il-23, Th17 cells experience “arrested development” leading to impaired function (40).

### Interleukin-17A

Interleukin-17A (IL-17A) is the first described member of the IL-17 cytokine family, which includes six members, IL-17A to IL-17F (41, 42).

Many IL-17A-producing cells have been reported, including T CD8^+^ cells (43), γδ T cells (44), and NK T cells (45); however, according to current knowledge, T CD4^+^ cells (Th17) are the major source of IL-17.

Although IL-23 has mainly been identified as the initiating factor for IL-17 expression from T cells (9, 46), Th0 cells do not constitutively express IL-23R, but they are still sensitive to IL-23 (47); in this context, it is reasonable to assume that IL-23R expression on these cells can be induced in the presence of other pro-inflammatory cytokines (48, 49).

Several findings clearly demonstrated that TGF-β and IL-6 are sufficient for Th17 differentiation *in vitro* and *in vivo*, in the absence of IL-23 (50–53).

Therefore, TGF-β, IL-6, and IL-21 seem to activate T lymphocytes and promote the initial differentiation of Th0 into Th17 cells, conferring responsiveness to IL-23 (50, 51, 54–60), which is a crucial step for Th17 cells stabilization and expansion (61).

Conversely, increased TGF-β levels coupled with the absence of inflammatory cytokines inhibit Th17 differentiation (62), as well as the common inhibitors of Th17 commitment (IFN-γ, IL-4, IL-25, IL-27) (54–56, 63–67).

Finally, IL-17-producing cells have been shown to express a wide range of heterogeneous cytokines such as IL-17A, IL-17F, IL-26 (62), and other proinflammatory mediators including IL-22, IL-21,IL-6, TNF-α, granulocyte colony-stimulating factors (GM-CSF), and chemokines (e.g., CCL20, CXCL8, CXCL1, CXCL10) (9).

### IL-17 Receptor

IL-17 receptor (IL-17R) is expressed on many cell types, including epithelial cells, B and T cells, fibroblasts, monocytic cells, and bone marrow stroma (68).

IL-17 signaling activates nuclear factor κB (NFκB) activator adaptor protein (ACT1), which in turn acts on mitogen-activated protein kinases (MAPKs), including p38MAK (69), c-jun N-terminal kinase (JNK), extracellular signal-regulated kinase (ERK), Janus kinase (JAK), signal transducer and activator of transcription (STAT), phosphoinositol 3 kinase (PI3K) and induces several pro-inflammatory cytokines (IL-1β, IL-6, TNF-α, CCL2), antimicrobial peptides (β-defensin), and matrix metalloproteinases (69–71).

In health conditions, IL-17 is one of the main contributors to the host defense against microbial infections (68, 72–74). Of note, the IL-17 pathway regulates antifungal immunity, in human and mice, inducing upregulation of proinflammatory cytokines, antimicrobial peptides and neutrophil-recruiting chemokines, which lead to limit fungal overgrowth (75).

Historically, CD4^+^ T cells have been involved in protecting against *Candida albicans* infection were found in Human Immunodeficiency Virus (HIV) positive patients (76, 77).

Subsequently, Th17 subset was identified as CD4^+^ T cells with reactivity to *Candida albicans* (37, 78).

Moreover, *ex vivo* studies on human T cells demonstrated that *Candida albicans* triggers Th17 cells which produce IL-17 and IFN-γ, but not IL-10. On the contrary, Staphylococcus aureus–activated Th17 cells produce IL-10, which can limit the immune system responses. These different responses could derive from the presence, in the priming phase, of different cytokine environments induced by each microbe (79).

The specific role of IL-17 in protection against the fungus *Candida albicans* was confirmed by evidence that IL-17RA deficient mice or mice and humans with defects along the IL-17 signaling pathway, were susceptible to systemic *Candida albicans* infection (80–83).

Major roles of IL-17 include the promotion and initiation of chemotaxis and the recruitment and activation of neutrophils in inflamed tissues (71, 84, 85). Among its pleiotropic effects, the enhancement of angiogenesis (86) and the tissue remodeling through the production of angiogenic factors and matrix metalloproteases are worthy of note (87). IL-17 works in synergy with TNF-α causing release of the IL-6, TNF-α, and IL-1β, in order to amplify the multifaceted and complex inflammatory process (88).

**Figure 2:**
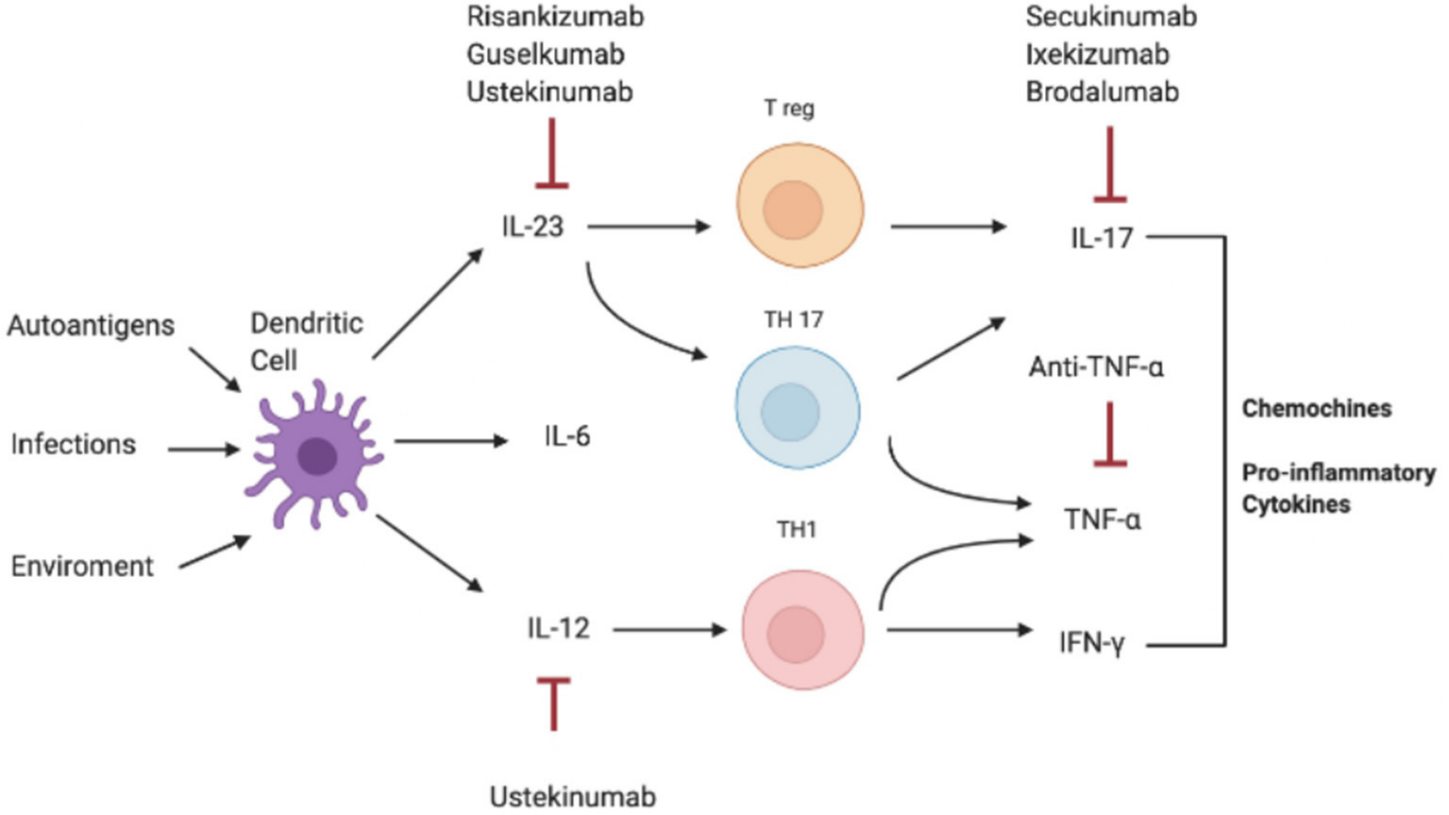
Pathogenesis of IL-17-correlated disease and different targets of therapy. IFN-γ, interferon-γ; IL, interleukin; Th, T helper; TNF-α, Tumor necrosis factor.

Consistently, increased serum and tissue levels of IL-17 have been widely reported in inflammatory condition such as IBD, MS, and arthritis, compared to a non-pathological setting where IL-17A levels are extremely low or undetectable in human sera (Figure 2) (3, 10, 89–92).

### Ankylosing Spondylitis (AS)

Ankylosing Spondylitis (AS) is the prototypical subset of SpA characterized by a predominant axial involvement (93). As a result, sacroiliitis is the clinical hallmark of disease and its identification through the most sophisticated imaging techniques, such as high-field magnetic resonance imaging (MRI), is extremely important in order to achieve an early diagnosis that can prompt a rapid treatment administration. The new classification criteria published by the Assessment in Spondylo-Arthritis international Society (ASAS) in 2009 have included MRI as the gold standard technique to identify active sacroiliitis, consisting in bone marrow oedema and osteitis, even in patients that have not developed radiographical signs of disease (94). According to these criteria, the presence of alterations (both in standard radiography or MRI) coupled with clinical, genetic and laboratory data, allow the classification of AS patients into radiological axial SpA (r-axSpA) or non-radiological axial SpA (nr-axSpA) (95).

New bone formation, determined by chronic inflammation involving the spine, leads to vertebral ankylosis, severe chronic pain and disability as major consequences of disease progression.

The beginning of the inflammatory process in AS relies on a complex, multifactorial interplay between genetic, epigenetic and environmental factors associated with a dysregulated immune response. The current understanding of AS pathogenesis suggests that the IL-23/IL-17 axis acts as the major driver in disease development, even if type 17 response could not entirely elucidate the mechanisms behind this rheumatic disease (96).

Genetics and epigenetics play a pivotal role in the pathogenesis of AS; siblings of AS patients have a higher risk of developing the disease and a high degree of concordance in twins is observed, 50–63% in monozygotic and 13–20% in dizygotic twins, respectively (97). The strongest genetic association is with the allele human leukocyte antigen B27 (HLA-B27) of the Major Histocompatibility Complex-I (MHC-I) gene, located on chromosome 6 (98).

Several theories have emerged to explain the possible pathomechanism related to HLA-B27, AS starting, and subsequent dysregulated activation of the IL-23/IL-17 axis via Th17 cells (99). Modifications in the shape of HLA-B27 affect the protein binding domain impairing both the antigen presentation process and the correct folding of the HLA-B27 molecule (100). The altered binding domain may lead to the presentation of self-peptides to cytotoxic CD8^+^ T cells that in turn give rise to a pathological autoimmune response (101). On the other hand, the unfolded protein response (UPR) theory postulates that unconventional HLA-B27 variants homodimerize instead of heterodimerize; the misfolded proteins accumulate in the intracellular compartment triggering endoplasmic reticulum stress and increasing IL-23 production (102). In fact, heterodimers expressed on APC surface directly interact with cell receptors on a wide range of immune cells such as NK, monocytes, B cells and promote a significant impact on Th17 stimulation (103). In particular, the aberrant expression of the allele of the HLA B 27 in spondylitis, could act by binding cells that contain a natural killer receptor for HLA B 27 homodimer, named killer cell immunoglobulin-like receptor 3DL2 (KIR3DL2), determining IL-17 production (104).

HLA-B27 alone accounts for almost the 25% of AS heritability and genetic-wide association studies (GWAS) have identified multiple genetic loci linked to disease pathogenesis (105, 106). In particular, single nucleotide polymorphisms (SNPs) in genes coding for aminopeptidases expressed in the endoplasmic reticulum (ER), such as ERAP1 and ERAP2, were identified in the past decade. These proteins trim peptides in the ER so that these molecules get to the right length, usually between 8 and 10 amino acids, to be presented by MHC-I molecules (107). Mutations in ERAP enzymes are supposed to lead to the formation of the so called “arthritogenic peptide” which, through mechanisms related to molecular mimicry, triggers immune cells to react against self-antigens located at joint and enthesal sites (108). ERAP1 and HLA-B27 effects are linked in an epistatic way, meaning that ERAP1 mutation effects are only observed in HLA-B27 positive patients (109).

SNPs directly affecting the IL-23/IL-17 axis were described in AS and further stress the importance of this pathway. The most relevant are located in the genes coding for IL-23R and STAT3 and TYK2, which are downstream targets of IL-23 signaling (110, 111).

However, it is not entirely clear how this polymorphism is responsible for pathogenesis, but this probably alters the amino acid sequence of the intracellular portion of IL-23R, and it could have a secondary regulatory function; indeed, protective alleles have also been identified in IL-23R, and some studies have detected polymorphisms in the IL-23R as a strong protective genetic factor (112).

The fundamental role played by the IL-23/IL-17 axis comes from several lines of evidence depicting a clear increase in IL-23 and IL-17 levels in the sera of AS patients (113). This observation is coupled with the increased number of Th17 cells in peripheral blood from AS patients (114). In this regard, the most intriguing and recent theories suggest that the interface between environment and immune system in AS can be represented by the gut epithelial barrier, where IL-17A exerts its functions in maintaining mucosal immunity and barrier functions (115). The intimate relationship between the articular disease and the gut is underlined by the detection of subclinical inflammation in up to 70% of AS patients on endoscopic examination, as well as on histological samples (116) and in about 10% of these occurs a clinical IBD suggesting a pre-clinical stage of IBD (115). Even bone marrow oedema in sacroiliac joints was correlated to gut inflammation, as emerged from the analysis of the Ghent cohort (117).

At gut level the huge number of adherent and invading bacteria, known as human microbiota, may be perturbed leading to both quantitative and qualitative alterations that affect the integrity of the gut-epithelial and vascular barriers in accordance with the “joint-gut axis theory” (118). The “leaky gut” allows the translocation of bacteria-derived peptides and primed immune cells to the interstitium and then to the bloodstream, eliciting a systemic abnormal inflammatory response. The derangement of the gut interface has been related to the alteration of the tight junction system and an increase in zonulin level was retrieved both in gut epithelium and peripheral blood (119). In AS patients dysbiosis was evidenced in comparison to healthy individuals and increased level of IL-23 are found in patients' gut and in particular in the terminal ileum (120). Several cell populations involved in epithelial immunity and joint/enthesal inflammation, such as Th17, ILC, γδ T cells, and mucosa-associate invariant T (MAIT) cells, as well as cells involved in mucosal homeostasis, such as Paneth cells, produce IL-23 at intestinal level. ILC3 are expanded in gut and differentiate upon IL-17 and TNF-α stimulation becoming an important source of IL-23 and IL-17 (121). Among the intraepithelial lymphocyte (IEL) compartment γδ T cells are the most represented population, accounting for approximately the 50% of IEL; on the other hand, they represent only the 3–5% of circulating T cells. Once activated with IL-23 these cells produce IL-17 (44). Their number was found increased in gut and γδ T cells obtained from AS patients show hyper-responsiveness to IL-23, due to IL-23R hyper-expression, with consequent discharge of higher amount of IL-17 when stimulated (122).

According to the gut-joint axis theory the intestinal activation of different immune cell subsets, among the ones above described, followed by their recirculation in blood may lead to their final localization in joint and enthesis where the inflammatory process is carried out. Intestine seems to be the major site of IL-23 production and is also the site where it acts most. Even gut- derived IL-17 producing ILC3 were found expanded at bone marrow, joint and peripheral blood level. ILC3 are also characterized by a significant expression of an integrin that regulates intestinal T cells homing, called α4β7. Moreover, the receptor of this integrin (named MADCAM1) was found upregulated in the gut and in bone marrow of AS patients, suggesting a chemoattraction process of ILC3 at inflammation site (121). Cuthbert et al. described resident γδ T cells at spinal enthesis where they contribute to IL-17 production in both IL-23 dependent and independent ways. In fact, a subpopulation of these cells lacking IL-23R was proven to produce IL-17 (123). In addition, IL-17 producing MAIT cells were found elevated in AS patients both in blood and synovial fluid (124, 125). To add more pieces to the already complex puzzle of IL-17 producing cells, a new population of CD8^+^ T cells able to produce IL-17, named Tc17 cells, was described and was found increased in both peripheral blood and synovial fluid of AS patients (126). Other possible sources of IL-17 that deserve deeper investigation are tissue-resident memory T cells (TRM), mast cells and CD3^−^CD56^+^NK cells (127, 128). In axial tissues IL-23 producing cells are macrophages and DC while IL-17 sources are myeloid cells as neutrophils (129, 130).

Animal models have supported the actual knowledge on the role of the IL-23/IL-17 axis, even if human disease appears far more complex and no animal model can comprehensively elucidate it. Transgenic HLA-B27 rat model was used to demonstrate the importance of the HLA-B27, gut microbiota and Th17 cells in the pathogenesis of SpA (131). These rats, grown in germ free condition do not develop SpA (132). Several mice models even exist and have contributed to the unraveling of the pivotal role played by IL-23 and IL-17 in activating T cells and driving disease development via type 17 immunity (133).

Taken together, the above resumed evidence underlines the importance of this axis in inducing and sustaining the multifaceted inflammatory process depicted in SpA, making it a central therapeutic target.

Advances in understanding the pathogenesis of SpA have prompted the development of biologic drugs designed to inhibit the IL-23/IL-17 axis. In fact, for more than 15 years, TNF-α inhibitors were the only biologic treatment available and, despite an initial great success in SpA management, it came out clearly that almost 40% of patients failed to reach a significative response. The new therapeutic agents interfering with the IL-23/IL-17 axis can be divided into monoclonal antibodies directly targeting IL-17, IL-23 or their receptors and small molecules inhibiting the intracellular pathways triggered by these cytokines (134).

Among monoclonal antibodies targeting IL-17, Secukinumab and Ixekizumab, respectively a fully human monoclonal IgG1/k antibody and a humanized IgG4 monoclonal antibody, are the only two molecules already marketed for SpA.

The MEASURE trials demonstrated the superiority of Secukinumab against placebo in providing sustained efficacy in relieving signs and symptoms of AS as well as in granting a good retention rate, as demonstrated in the 5-years extension study (135, 136). In the 2-years follow up in the MEASURE 1 trial no radiographic progression was evidenced in the 80% of patients included (137).

Ixekizumab was licensed for r-axSpA treatment in 2019 and for nr-axSpA in 2020, the COAST-V trial demonstrated a superior response rate in ASAS40 score at week 16 over placebo (138).

Several trials aimed to assess the therapeutic value of other IL-17 inhibitors, such as Netakimab and Bimekizumab, are currently ongoing (139, 140). The randomized controlled trial on Brodalumab, a humanized IgG2 monoclonal antibodies that binds IL-17R was discontinued because of the occurrence of high suicide ideation in the active group (141).

Up to date, no IL-23 targeting drug has been proven effective for AS (142, 143).

New treatments for AS are small molecules inhibitors that target intracellular proinflammatory pathways, as those triggered by cytokine stimulation. Among targeted synthetic DMARDs (tsDMARDs), JAK-inhibitors stand out as the most promising treatment (144). In particular, Tofacitinib, a pan-JAK inhibitor, Filgotinib and Upadacitinib, both JAK1 inhibitors, were shown to be superior to placebo in axSpA (145–147).

The inhibition of PDE4 through the small molecule Apremilast failed to achieve the primary end-point in the specifically designed phase 2 trial including 490 AS patients (148).

Future perspectives to implement the therapeutic options for AS patients include broader anti-inflammatory approaches with multi-cytokine blockade to overcome the inhibition of a single pathway, for example targeting simultaneously TNF-α and IL-17, as in an ongoing study in PsA (149).

### Psoriatic Arthritis

Psoriatic arthritis (PsA) is a chronic, immune-mediated, inflammatory disease dominated by a heterogeneous phenotype that mainly affects peripheral and axial joints, entheses, skin, and nails, leading to juxta-articular new bone formation, bone erosions and abnormal keratinocyte proliferation (31, 150).

Interactions between genes and environmental triggers, including infections, trauma, stress, obesity and smoking are recognized as crucial for the onset of the autoimmune process in PsA. Furthermore, the disruption of the gut microbiota composition in PsA patients is supported by consolidate evidence (151, 152).

The main effector cells of the inflammatory cascade, both in joints and in plaques of patients with PsA, are DCs, macrophages, NK cells (153, 154), mast cells, neutrophils (155, 156), γδ T cells (157), T CD4^+^ and T CD8 ^+^ cells.

All the ones above described have a predominant IL-17 secretory phenotype (41, 158), defined by the production of cytokines such us IL-17, IL-22, and TNFa; however, T CD4^+^ cells, as the major source of IL-17, are considered the cornerstone in the pathogenesis of psoriasis (157, 159).

Specifically, DC-derived cytokines, IL-23 and IL-12, drive the differentiation of distinct Th17 and Th1 cells, which are known to be implicated in the pathogenesis of PsA. Activated T cells move from the circulation to the target organs, and chronic inflammation occurs in the skin and joints.

Accordingly, increased numbers of Th17 cells were detected in the blood and affected skin of patients with psoriasis and in the blood and synovial fluid of patients with PsA. Furthermore, the assessment of the expression of IL-23, IL-17, and their related receptors in psoriatic skin lesions and inflamed synovium supports the concept of IL-23/IL-17 axis as a driving force of immune inflammation in psoriasis (160–164) PsA synovitis is characterized by significant infiltration of mononuclear cells, T and B cells, vascular proliferation and hyperplasia of synovial lining cells, similar to the pathological changes observed in RA (165). Additionally, ectopic lymphoid tissues were frequently found in PsA synovial membrane with microanatomical features for germinal center formation, capable of antibody production (166). The role of B cells in PsA is still elusive; a recent study reported that autoantibodies against a peptide sharing sequence homology with skin and entheseal autoantigens were detected in 85% of patients with PsA (167).

Several findings have clearly demonstrated the influence of IL-17 on bone metabolism. Despite the direct effects of IL-17 on osteoclasts, induction of osteoclastogenesis is mediated by the production of matrix metalloproteinases by macrophages and the activator of the NF-κB ligand receptor (RANKL) presented by osteoblasts (168–170).

Skin lesions from patients with psoriasis exhibit epidermal hyperplasia and infiltration with neutrophils, T CD4 ^+^ and T CD8 ^+^ cells, B cells, dendritic cells and mast cells, type 3 innate lymphoid cells (ILC3) and γδ T cells (171–174).

In contrast, IL-22, totally absent in synovial tissue, is highly expressed in entheses, where it promotes entheseal and periosteal bone formation through STAT3 activation, explaining the formation of enthesophyte and juxta-articular bone, hallmarks of PsA (175, 176). In skin lesions IL-22 drives keratinocyte hyperproliferation via STAT3 signaling (177) and prompts epithelial cells to release chemokines, such as IL-8 (178, 179), a key factor in neutrophil recruitment in psoriatic lesions and stimulates keratinocytes to secrete antimicrobial peptides (180, 181), preventing skin lesions from becoming infected (182).

In inflammation, Th17 cells also produce interleukin 9 (IL-9), which in turn induces the differentiation of Th17 cells and potentiates the suppressive effect of regulatory T lymphocytes via activation of STAT3 and STAT5. Our group demonstrated that IL-9 overexpression and T helper type 9 (Th9) polarization occur in the synovial tissue and peripheral blood of PsA patients. Furthermore, clinical improvement after treatment with TNFi and ustekinumab was associated with a significant reduction in circulating Th9 cells (183, 184).

Up to date, a wide range of therapeutic approaches have been proposed for PsA, depending on disease severity, including disease-modifying anti-rheumatic drugs (DMARDs) such as methotrexate; anti-tumor necrosis factor α (anti-TNF-α) agents, phosphodiesterase 4 (PDE4) inhibitors, IL-17 and IL-12/IL-23 inhibitors (185, 186).

The central contribution of IL-23/IL-17 axis in both PsA and psoriasis is confirmed by the efficacy of biologics neutralizing IL-17 or IL-23/IL-12 (187) as well as the effectiveness of TNF-α inhibition dependent on down-regulation of IL-17 pathway genes (188–190).

To date, several monoclonal antibodies have been approved for the treatment of PsA and can be introduce following the failure of non-steroidal anti-inflammatory drugs (NSAIDs) and/or conventional DMARDs, or an anti-TNF-α agent.

Ustekinumab is a human IgG1 monoclonal antibody that binds to the p40 subunit, shared with IL-12 and IL-23, and blocks downstream events of both the IL-12 and IL-23 signaling cascade through inhibition of IL-12Rβ1 binding (191). Ustekinumab has produced consistent and sustained clinical efficacy in two phase three clinical trials in PsA, PSUMMIT-1 and PSUMMIT-2, with data out to 52 weeks, and no new safety signals. PSUMMIT-1 included patients with active PsA despite conventional therapy who were all naïve to anti-TNF-α agents, whereas PSUMMIT-2 also included anti-TNF-α experienced patients (192).

Secukinumab, is a human IgG1κ monoclonal antibody that binds to IL-17A neutralizing its interaction with IL-17 receptors. It is currently approved in several countries for the treatment of PsA (193) and, in two phase 3 trials FUTURE1 and FUTURE 2, secukinumab has provided sustained improvements in disease signs and symptoms, assessing reduced radiographic progression in patients with active PsA through 2 years of therapy (194).

Ixekizumab is a humanized IgG4 monoclonal antibody targeting IL-17A, approved for the treatment of moderate-to-severe plaque psoriasis, active PsA, and active AS. Two phase three trials (SPIRIT-P1 and SPIRIT-P2) demonstrated that treatment with ixekizumab improved joint and skin disease compared to placebo (195, 196).

In addition, the SPIRIT-H2H study confirmed the superiority of ixekizumab over adalimumab in patients with Psa and inadequate response to csDMaRDs (197).

Over the past decade, the development of Janus kinase inhibitors (JAK inhibitors) has emerged as a new therapeutic option in autoimmune diseases, including PsA. The rationale of their use is suggested by the observation that the blockade of JAK receptor downregulates the production of the cytokines (TNF-α, IL-17, IL-6, IL-23) involved in the pathogenesis of PsA.

Tofacitinib is an orally administered inhibitor of predominantly JAK1 and JAK3, with functional selectivity to JAK2, used for the treatment of RA. Interestingly, its efficacy in managing treatment-resistant disease and ameliorating enthesitis, dactylitis, and radiographic progression has been reported. Consistently with previous observations, Tofacitinib could provide an alternative approach for PsA patients with inadequate response to DMARDs (198, 199).

Finally, Guselkumab, a monoclonal antibody targeting IL-23 via IL-23 p19 subunit, was recently approved for the treatment of PsA in adults with an inadequate response or intolerance to DMARDs therapy. Results for the use of Guselkumab derive from phase three clinical trials, DISCOVER-1 and DISCOVER-2, which demonstrated significantly better clinical and radiographic outcomes among PsA patients treated with the IL-23 inhibitor compared with placebo group.

Participants treated with Guselkumab achieved 20% improvement in American College of Rheumatology (ACR) response criteria at week 24 at rates of 52% in DISCOVER-1 and 64% in DISCOVER-2, whereas placebo-treated patients had rates of 22 and 33%, respectively (200, 201).

### Rheumatoid Arthritis (RA)

Rheumatoid arthritis (RA) is a systemic autoimmune disease that primarily affects synovial joints, accompanied by systemic inflammation and production of autoantibodies (202).

The synovitis in RA is characterized by an inflammatory infiltrate, consisting of leukocytes such as T and B cells, macrophages, granulocytes and dendritic cells, together with a synovial milieu dominated by proinflammatory cytokines and chemokines (203).

For a long time, RA was considered a Th1 dependent disease, until a significant amount of research in RA patients and experimental mouse models suggested that Th17 cells may play a central role in the pathogenesis of RA.

IL-17 is involved in both early and established RA disease, promoting activation of fibroblast-like synoviocytes (FLS), osteoclastogenesis, recruitment and activation of neutrophils, macrophages and B cells (204).

Synergism between IL-17 and TNF-α has been shown to activate the production of pro-inflammatory mediators, such as IL-1β, IL-6, IL-8, PGE2, and matrix metalloproteinases (MMPs), promoting progression of early inflammation toward a chronic arthritis (205, 206).

The differentiation of osteoclasts is induced significantly in the presence of IL-17 either directly (207), or indirectly, through upregulation of RANKL.

By the late 1990s studies had already shown that IL-17 expression had increased in the joint of RA patients compared to healthy individuals or osteoarthritis (OA) patients (89).

More recently, an increased proportion of chemokine receptor CCR6^+^ Th17 cells has been described in the peripheral blood of treatment-naive patients with early RA (208), and higher frequencies of Th17 cells have been detected in the synovial compartment of RA patients, compared to OA patients (209).

Moreover, Th17 cells were associated with clinical parameters, such as disease activity score 28 (DAS28), C-reactive protein (CRP) levels and presence of anti-citrullinated protein antibodies (ACPAs), highly specific for RA (210, 211).

Direct evidence obtained from experimental mouse models confirms the critical role of the IL-23/IL-17 axis in the pathogenesis of arthritis. IL-23p19-deficient (Il23a^−/−^) mice were protected against the development of collagen-induced arthritis (CIA), a mouse model of RA. IL-17-producing CD4^+^ T cells were absent in the Il23a^−/−^ mice despite normal induction of IFN-γ-producing CD4^+^ Th1 cells (212).

Interplay between IL-23 and IL-17 production may be a critical immune pathway and a potential therapeutic target for a range of inflammatory arthritis.

Antibodies against IL-17 (Ixekizumab and Secukinumab) or IL-17R (Brodalumab) have been tested in RA patients (213–216). In a phase I RCT of RA patients treated with oral DMARDs, the addition of Ixekizumab improved RA signs and symptoms and disease activity scores such as DAS28, compared to placebo (213). This improvement was confirmed in a phase II study in which Ixekizumab was administered to patients who were naive to biological therapy or resistant to TNF-α inhibitors (217).

In a phase II study enrolling RA patients with inadequate response to methotrexate, greater decreases in DAS28 were observed with Secukinumab than with placebo (214).

Furthermore, patients with active RA who did not respond to DMARDs showed improvements after long-term treatment (52 weeks) with 150 mg of Secukinumab, with ACR50 rates increased from 45% to 16 to 55% at week 16 at week 52 (215). Conversely, Brodalumab showed no evidence of clinical benefit in RA patients in a phase Ib study (213).

In addition, the efficacy of Ustekinumab, a human anti-IL-12/23 p40 monoclonal antibody and human anti-IL-23 monoclonal antibody, Guselkumab, has been evaluated in patients with active RA not responsive to methotrexate therapy. However, no significant clinical improvement was recorded compared to the control group (218).

Recently, the double blockade of IL-17 and TNF-α has been studied using ABT-122, a variable double domain Ig that targets human TNF-α and IL-17 (219). A phase II study showed that there was no significant difference in the ACR20 response at week 12 by the double inhibition of IL-17 and TNF-α compared to treatment with only anti-TNF-α (220). Lastly, double blockade of IL-17A and IL-17F using Bimekizumab in RA patients with inadequate TNF-α response resulted in a greater reduction in DAS28-CRP at week 20 compared to anti-TNF-α inadequate response plus placebo group (221).

### Sjögren's Syndrome (SS)

Sjögren's syndrome (SS) is a systemic autoimmune disease characterized by the lymphocytic infiltration into the exocrine glands, mainly salivary and lacrimal glands, and other tissues (222).

The role of the adaptive immune system in the pathogenesis of SS is supported by the presence of ectopic germinal centers in almost 25% of the patients, which promote local expansion of antigen-specific B cells, production of autoantibodies and hypergammaglobulinemia, as well as increased risk of developing non-Hodgkin lymphoma (NHL) (223, 224).

In this regard, the interaction between CD4^+^ T cells and B cells appears to be a key step in the development of the disease (222).

Although SS has historically been considered a Th1-driven disease, subsequent studies have revealed that, in addition to Th1 cells, several subgroups of CD4^+^ T cells are involved, follicular T helper cells (Tfh) and Th17. In healthy subjects, Th17 cells play an important role at mucosal barriers and are involved in immune responses. In SS, Th17 cells may be activated by dendritic cells in lymph nodes draining the salivary and lacrimal glands by the production of cytokines as TGF-β and IL-23. In the later phases of the disease, naïve T cells can also be polarized locally in Th17 cells by APC and cytokines as IL-6 and TGF-β (225). IL-23 production by macrophages is also important for maintenance and expansion of Th17 cells by STAT3 activation (226). The main effector cytokines of Th17 cells are IL-17 and IL-22. Recent studies reported that IL-17 protein and mRNA are present within lymphocytic infiltrates of minor salivary gland (MSG) tissue of SS patients.

Furthermore, IL-17 mRNA levels in MSG biopsies were related to the degree of inflammation (226–228).

Along with IL-17, the expression of IL-23 and IL-22 was also increased in the inflamed salivary glands of SS patients. IL-17 and IL-22 secretion in the exocrine glands promote inflammation and the induction of matrix metalloproteinases which may cause acinar damage (229).

Moreover, the presence of Th17 cells in SS infiltrate has been hypothesized to be crucial in B cell activation and formation of germinal centers within glands (230).

The IL-23/IL-17 axis also plays a role in the symptoms development, as confirmed by the increased levels of IL-17 and IL-6 protein and mRNA in tears and saliva from SS patients compared to non-SS controls. Additionally, the expression of Th17-associated cytokines correlated with ocular surface parameters, such as Schirmer I Test, break up time (BUT) and corneal fluorescent staining (CFS) (231).

A recent study showed that serum IL-17F levels were significantly increased in SS patients and were associated with high levels of autoantibodies and increased EULAR SS disease activity index (ESSDAI), compared to IL-17A, suggesting the possibility of several pathogenetic roles played by different IL-17 family members (232).

The role of the IL-23/IL-17 pathway in the pathogenesis of SS has been also supported by data from animal models. In C57BL/6.NOD-Aec1Aec2 mouse, a model of spontaneous SS, genetic ablation of IL-17 reduced lymphocytic infiltration and restored glandular function, especially in female animals (233).

In addition, adoptive transfer of Th17 cells induced the development of experimental SS (ESS) in immunized IL-17 knockout mice (234).

Even if Th17 cells are the main source of IL-17 in SS, γδ T cells, NK cells, ILCs and CD8^+^ T lymphocytes can also produce IL-17 (68). However, recent evidence has shown that CD4^−^ CD8^−^ (double negative, DN) T cells and mast cells can also participate in local IL-17 production in SS (235).

Rituximab treatment demonstrated a significant reduction in IL-17 expression in the salivary glands of SS patients, while factors that are important for maintenance of Th17 cells, as STAT3 and IL-23, are likely not affected (228).

Despite the increase in Th17 cells in SS is well-established, data on the amount of T reg cells are still controversial. Th17 and Treg cells are counter-regulated. IL-6 promotes Th17 differentiation by inhibiting T reg generation (51), while IL-2 acts in the opposite way (236). Since reduced IL-2 levels could underline Th17 upregulation (237), the effects of administration of recombinant IL-2 have been studied in SS. Reduction of glucocorticoid and Hydroxychloroquine (HCQ) in patients treated with IL-2 and restored Th17/Treg balance have been reported (238).

Regarding IL-6 inhibition, as the IL-6 signaling is important for the IL-17 cells differentiation, an effect of tocilizumab on Th17 cells is probable. However, the results of a randomized placebo-controlled study showed that Tocilizumab had no impact on the main symptoms of SS, although improvements in joint involvement were observed (239).

Long-term efficacy of Ustekinumab, a human monoclonal antibody directed against the p40 protein subunit shared by IL-12 and IL-23, in a patient suffering from psoriasis and SS has been recorded on both the cutaneous and joint component (240). However, the inhibition of IL-17 achieved by systemic administration of Secukinumab did not affect the severity of the dry eye (241). Given the pilot role of the IL-23/IL-17 axis in SS pathogenesis, further investigations are needed to confirm the selective blockade of Th17-associated cytokines as a potential therapeutic target.

### Systemic Lupus Erythematosus (SLE)

Systemic lupus erythematosus (SLE) is a prototypic systemic autoimmune disease with multiple immunological abnormalities including dysregulation of both T and B lymphocytes and production of autoreactive antibodies directed toward nuclear self-antigens, with immune complex formation and tissue damage (242). A wide number of cytokines is involved in disease pathogenesis. Recently, the role of the IL-23/IL-17 axis has emerged in SLE and has been investigated either in humans or mice. Higher serum levels of IL-17 and IL-23 as well as increased number of Th17 cells has been demonstrated in patients with SLE compared with healthy controls (243, 244). Moreover, T cells that express IL-17 were found within infiltrates in kidney biopsies of patients with active lupus nephritis and significant proportion of these cells are double negative (DN) T cells, which are also expanded in the peripheral blood of patients with SLE, produce significant amounts of IL-17 and contribute to the disease pathogenesis (245). In a murine model of lupus, Zhang et al. detected DN T cells expressing high levels of IL-17A in the kidneys of mice with active nephritis. Interestingly, the lymphocytes isolated from these lupus-prone mice progressively express higher levels of IL-23 receptor as their disease worsens. Treating these lymphocytes *in vitro* with IL-23, they induce nephritis when transferred to non-autoimmune, control mice. Additionally, the kidneys of these recipient affected mice showed significant Ig and complement deposition in the glomeruli. This finding suggests that IL-23 also promoted an autoimmune B cell response. These data added to previous evidence indicate that DN T cells provide excessive help to B cells, resulting in abnormal production of pathogenic autoantibodies in SLE (246).

Consistently with previous findings, Chen et al. found that circulating Th17 frequency correlated with SLE activity, in particular with Systemic Lupus Erythematosus Disease Activity Index (SLEDAI) and histological activity index in 24 lupus nephritis (LN) patients (247).

The link between IL-23/IL-17 axis and LN is also supported by the observation that high serum levels of IL-23 and IL-17 at baseline predict an unfavorable histopathological response and British Isles Lupus Assessment Group (BILAG)-non-responders had high IL-23, indicating that a number of LN-patients has a Th-17 phenotype that may influence response to treatment.

In addition to LN, high serum levels of IL-23 and Th17 cells are closely related to other SLE manifestations, including vasculitis, serositis, lymphopenia, central nervous system and cutaneous involvement and the production of autoantibodies (antinucleosome antibodies, antiphospholipid antibodies, and anti-SS-B/La antibodies) (244, 248–250).

Taken together, these findings suggest that Th17 cells are a promising therapeutic target for SLE.

Previous studies have shown that Hydroxychloroquine (HCQ), an essential drug for the treatment of SLE, can inhibit Th17 cell differentiation and production (251).

In a phase two trial of 102 adult patients with active SLE, the addition to standard-of-care treatment of Ustekinumab, a human IgG1k monoclonal antibody targeting both the IL-12 and IL-23 cytokines, resulted in better efficacy in clinical and laboratory parameters than placebo. In particular, 37 (62%) of 60 patients in the Ustekinumab group and 13 (33%) of 42 patients in the placebo group achieved a SLE disease activity index 2000 responder index-4 (SRI-4) response (difference 28%, *p* = 0.006) (252).

Few data are available in literature about the anti-IL-17A antibody (Secukinumab). A case of 62-year-old female who presented with psoriasis vulgaris and refractory lupus nephritis successfully treated with Secukinumab was reported. Further studies are needed to test the efficacy of drugs targeting.

## Conclusion

This review provides an overview of the critical role played by the IL-23/IL-17 immune axis in a wide variety of inflammatory processes, and summarizes the current knowledge on cytokine milieu that regulates IL-17-producing cells. The growing body of evidence on the relevance of this intricate pathway in autoimmunity has allowed the implementation of treatment options in pathological conditions.

However, further studies are needed to clarify the complexity of IL-17 signaling, in order to allow the discovery of new potential therapeutic targets of inflammatory processes and the availability of cutting edge therapies designed on patients not responsive to standard treatments.

## Author Contributions

CS and SF: conceptualization, methodology, formal analysis, investigation, resources, data curation, writing—original draft preparation, writing—review & editing, and visualization. GGr and FC: methodology, formal analysis, data curation, and writing—review & editing. CR: software, methodology, formal analysis, data curation, and writing—review & editing. LL: formal analysis, data curation, and writing—review & editing. GGu: conceptualization, methodology, formal analysis, investigation, resources, data curation, writing—original draft preparation, writing—review & editing, visualization, supervision, and project administration. All authors had access to the study data and reviewed and approved the final manuscript.

## Conflict of Interest

The authors declare that the research was conducted in the absence of any commercial or financial relationships that could be construed as a potential conflict of interest.
